# A W-Shaped Self-Supervised Computational Ghost Imaging Restoration Method for Occluded Targets

**DOI:** 10.3390/s24134197

**Published:** 2024-06-28

**Authors:** Yu Wang, Xiaoqian Wang, Chao Gao, Zhuo Yu, Hong Wang, Huan Zhao, Zhihai Yao

**Affiliations:** 1 Department of Physics, Changchun University of Science and Technology, Changchun 130022, China; 2021200025@mails.cust.edu.cn (Y.W.); gaoc@cust.edu.cn (C.G.); yuzhuo@mails.cust.edu.cn (Z.Y.); hongwang@mails.cust.edu.cn (H.W.); 2019200015@mails.cust.edu.cn (H.Z.); 2 School of Physics and Electronics, Baicheng Normal University, Baicheng 137000, China

**Keywords:** deep learning, self supervised, neural networks, ghost imaging

## Abstract

We developed a novel method based on self-supervised learning to improve the ghost imaging of occluded objects. In particular, we introduced a W-shaped neural network to preprocess the input image and enhance the overall quality and efficiency of the reconstruction method. We verified the superiority of our W-shaped self-supervised computational ghost imaging (WSCGI) method through numerical simulations and experimental validations. Our results underscore the potential of self-supervised learning in advancing ghost imaging.

## 1. Introduction

Ghost imaging is a nontraditional imaging method that has the ability to capture object images in inaccessible environments. It is robust against interference and enable nonlocal image reconstruction, exploiting the features of second-order correlation functions [1,2,3,4,5,6]. The groundbreaking work of Pittman et al. demonstrated the feasibility of ghost imaging using entangled photons [7]. Ghost imaging provides a unique and promising alternative to conventional imaging techniques such as charge-coupled devices (CCDs) and complementary metal–oxide semiconductor (CMOS) cameras. It has numerous applications, including imaging through scattering media [8,9], color object imaging [10], and detection of moving objects [11,12].

Many methods to improve the practical use of ghost imaging (GI) have been developed, including differential ghost imaging (DGI) [13], normalized ghost imaging (NGI) [14], and alternating projection ghost imaging (APGI) [15]. These methods provide enhancement in the quality of ghost imaging, while computational ghost imaging (CGI) [16] has made experiments simpler. On the other hand, multiple data acquisitions are often required to achieve satisfactory imaging results. This is because the information collected by bucket detectors in each measurement is limited. For example, if a two-dimensional object has M pixels (M=ab, where *a* denotes the number of horizontal pixels, and *b* the of vertical ones), the number of required measurements N corresponds to a sampling rate β = N/M. Traditional ghost imaging often requires N to be much larger than M to obtain high-quality imaging, if random speckles are used for illumination. Compressed sensing ghost imaging [17] and deep learning ghost imaging [18,19,20] have been proposed to address these issues. Both methods have demonstrated their ability to achieve high-quality imaging at low sampling rates. However, the reconstruction algorithms based on compressed sensing ghost imaging are highly sensitive to background noise [17,21], which limits their practical applications. Deep learning ghost imaging combines the principles of ghost imaging with the capabilities of deep neural networks to achieve high-quality imaging at low sampling rates [18]. Deep learning ghost imaging has demonstrated significant performance in noise reduction [22,23,24,25,26,27,28,29]. In addition, it can be applied in various fields [30,31,32,33,34,35], but these deep learning methods require a lot of data and lengthy training, which is very time-consuming. In order to improve the efficiency of deep learning retrieval imaging, self-supervised learning approaches have been proposed [36,37,38]. These methods can produce high-quality images without the need to collect data.

In practical applications of ghost imaging, partial occlusion of the object under test may occur. Although previous studies have demonstrated that ghost imaging has some imaging capabilities for occluding objects [4], the method requires the distance between the target object and the obstacle to be sufficiently far, and the occluding object needs to be between the object under test and the bucket detector, which is not easily achievable in practical applications. Other related studies have focused on scattering media such as atmospheric turbulence [39,40], which also imposes certain limitations on practical applications.

The above method has certain advantages in ghost imaging with occluding objects, but there are still some unresolved issues, such as how to image nontransparent occluding objects and how to improve the efficiency of occluding-object ghost imaging. Therefore, addressing these issues remains a challenge, prompting us to develop a method called W-shaped self-supervised computational ghost imaging (WSCGI). This method, combining self-supervised deep learning techniques, quickly achieves high-quality imaging at low sampling rates. At the same time, it can also restore the parts obscured by opaque objects. Compared with other occluding-object ghost imaging methods, our approach is simple and practical and greatly improves the imaging efficiency.

This paper is structured as follows: Section 2 describes WSCGI, outlining its principles and methods. In Section 3 and Section 4, we discuss the effectiveness of WSCGI in restoring occluded objects using results from numerical simulations and experiments, respectively. Finally, Section 5 summarizes the main research findings and conclusions.

## 2. W-Shaped Self-Supervised Computational Ghost Imaging

**Figure 1:**
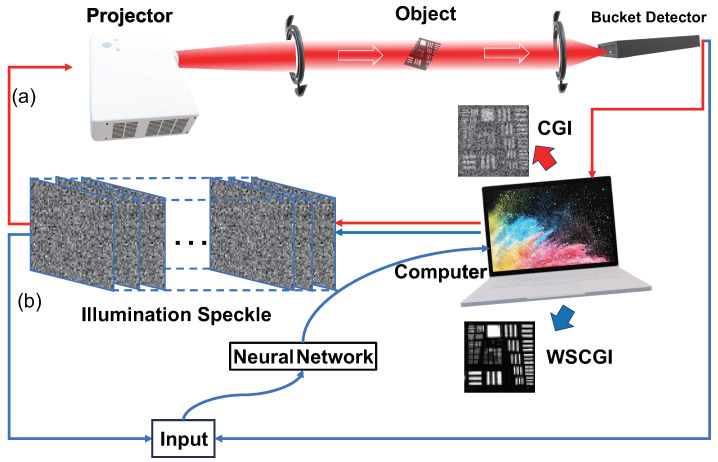
Schematic diagram of the experimental setup for computational ghost imaging and WSCGI: (**a**) computational ghost imaging; (**b**) WSCGI.

Figure 1a illustrates the setup of our computational ghost imaging experiment. At first, a series of random illumination speckle patterns were generated by the computer and loaded onto the projector. Then, the light passed through the object and was collected by a bucket detector. At last, the collected intensity signals were sent to the computer. The set of collected signals $B_{m}$ can be written as

(1) $$B_{m}=\;\int \int \;I_{m}\left(x,y\right)T\left(x,y\right)\;dx\;dy\;,$$

where $I_{m}\left(x,y\right)$ represents the speckle pattern of the mth measurement with m=1,2...,N, and N represents the total number of measurements. T(x,y) denotes the transmittance function of the object, and (x, y) denotes the coordinates of the target. By leveraging the principle of normalized ghost imaging, it is possible to effectively reduce noise interference, thereby obtaining high-quality images. This corresponds to

(2) $$T_{NGI}\left(x,y\right)=\langle B_{m}I_{m}\left(x,y\right)\rangle -\frac{\langle B_{m}\rangle }{\langle S_{m}\rangle }\langle B_{m}I_{m}\left(x,y\right)\rangle$$

where 〈...〉 denotes averaging over N measurements, and $S_{m}=\;\int \int \;I_{m}\left(x,y\right)\;dx\;dy$.

Although algorithmically optimized computational ghost imaging methods have achieved some degree of success, they still require a large number of measurements and demanding experimental conditions. Deep-learning-based ghost imaging methods can be employed, but they often rely on manually designed feature extractors and extensive labeled data, resulting in significant time consumption. The method is shown in Figure 1b.

**Figure 2:**
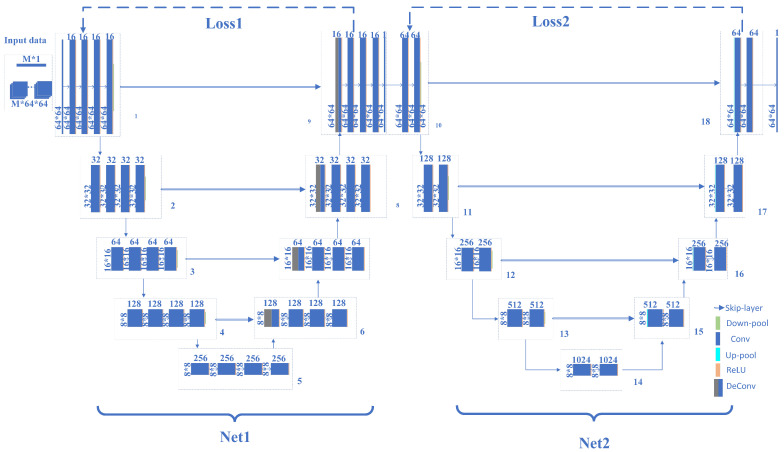
WSCGI network architecture diagram. The neural network consists of three main parts: (1) processing the input data using the NGI method to obtain low-quality images; (2) Net1, which is composed of convolutional layers, downsampling layers, deconvolutional layers, batch normalization, and the LeakyReLU activation function; (3) Net2, which is composed of convolutional layers, upsampling layers, downsampling layers, and batch normalization, utilizing the LeakyReLU activation function.

The input for the WSCGI network consists of signals from the bucket detector and computer-generated speckle patterns. Upon exploiting deep learning techniques, it autonomously learns image representations, eliminating the need for dataset preparation, and accomplishes the reconstruction of occluded objects. The neural network that we designed facilitates iterative refinement of the final results. In other words, we preprocess the input images to enhance their quality and the overall information they provide, before proceeding with occlusion recovery. Such a structure significantly enhances the probability and quality of occlusion imaging recovery. The network architecture of WSCGI is illustrated in Figure 2.

Our WSCGI network is made of three main parts: The first one handles network inputs, receives the bucket detector signals as well as the input speckle signals generated by the physical GI model, and computes the initial image input. The mathematical principles underlying this process are illustrated in Equation (2).

The second part is Net1, which is primarily responsible for enhancing the input image. Net1 consists of multiple convolutional and deconvolutional modules, ultimately yielding an output using a sigmoid function. The convolutional and deconvolutional modules include convolution layers, batch normalization (BN) layers, and LeakyReLU layers. The convolution kernel size is 5 × 5, with a learning rate of 0.01, an exponential decay rate of 0.9, and a decay step size of 100. Additionally, Net1 processes the entire image, thus needing more detail and structural information, and hence requiring relatively more network layers. By harnessing the inherent low sensitivity to natural signals and high resistance to noise of neural networks, we can effectively filter out abrupt components in degraded images by controlling the number of iterations. Mathematically, this can be expressed as

(3) $$\min_{\theta }\frac{1}{2}{\left\|T_{NGI}-f_{\theta }\left(z\right)\right\|}^{2}+\lambda R\left(\theta \right)\;,$$

where $T_{NGI}$ represents the low-quality input image, f(·) denotes the network framework, and θ are the network parameters. We incorporate total variation (TV) regularization as a regularization term (λR), where λ is the regularization coefficient. The image *z* is randomly generated, i.e., it does not contain any specific information learned by the network about the original image. By minimizing the mean square error term and the total variation regularization term in the loss function, we optimize the network parameters to achieve image enhancement. The total variation regularization term is also exploited to smooth the image and enhance edges, thereby reducing noise in the reconstruction process and preserving the information details of the image. Overall, Net1 can simultaneously achieve functions such as image denoising and edge enhancement.

The third component is Net2, tasked primarily with restoring the occluded parts of the image by further processing the output of Net1. The structure of Net2 is largely similar to that of Net1 and consists of four convolutional modules with downsampling layers, and four convolutional modules with upsampling layers. LeakyReLU is employed as activation function. The convolution kernel size is 5 × 5, with a learning rate of 0.01, an exponential decay rate of 0.9, and a decay step size of 100. Net2 primarily deals with occluded local regions, hence requiring the processing of more localized contextual information. Compared to Net1, it requires relatively fewer network layers, and the mathematical expression of its action may be written as follows:

(4) $$\begin{array}{c} E\left(\vec{P};{\vec{P}}_{0}\right)=\frac{1}{N}\sum_{i=1}^{N}{\left(P_{i}-P_{0i}\right)}^{2}\;, \end{array}$$

where $P_{0}$ represents the input image, *P* represents the predicted image, $P_{i}$ and $P_{0i}$ denote the *i*th pixel values of the predicted and input images, respectively, and *N* is the total number of pixels. The patching process aims at filling the occluded regions of the image while minimizing the discontinuities and distortions in the surrounding areas. To achieve this goal, we use the mean squared error as the loss function and minimize the objective function E(…), ensuring that the completed image $P_{i}$ closely matches the reference pixel values at each position. Upon employing gradient descent optimization algorithm, we iteratively update the parameters of the model, enabling the generated image to closely resemble the original image, thereby completing the image patching process.

In evaluating the performance of self-supervised learning network aimed at reconstructing images, various metrics are commonly employed. These metrics include peak signal-to-noise ratio (PSNR) [41], the structural similarity index (SSIM) [42], and the discriminator of generative adversarial networks (GANs) [43]. However, in the absence of original images, these methods may lack objectivity. To address this issue, we introduce the natural image quality evaluator (NIQE) [44], which is a assessment method that does not require a reference and is capable of automatically evaluating image quality. The NIQE was developed for accurately assessing image quality by analyzing local features to simulate the statistical properties of natural images. The evaluation formula is written as follows:

(5) $$\begin{array}{c} D\left(\mu_{1},\mu_{2},\Gamma_{1},\Gamma_{2}\right)=\sqrt{\left({\left(\mu_{1}-\mu_{2}\right)}^{T}{\left(\frac{\Gamma_{1}+\Gamma_{2}}{2}\right)}^{-1}\left(\mu_{1}-\mu_{2}\right)\right)} \end{array}$$

where *D* denotes the final score obtained from the NIQE, $\mu_{1}$ and $\Gamma_{1}$ are the mean vector and the covariance matrix obtained within the NIQE model, and $\mu_{2}$ and $\Gamma_{2}$ are the mean vector and the covariance matrix of the input image. Ultimately, we employed the NIQE score of the image to assess its quality, ensuring that our network effectively identified the highest-quality images during the training process.

## 3. Simulations

The image size used in the numerically simulated experiments was 64 × 64 pixels. In the experiment, the images were divided into complex images and simple binary images. The sampling rate β refers to the ratio between the number of sampling points and the total number of pixels.

**Figure 3:**
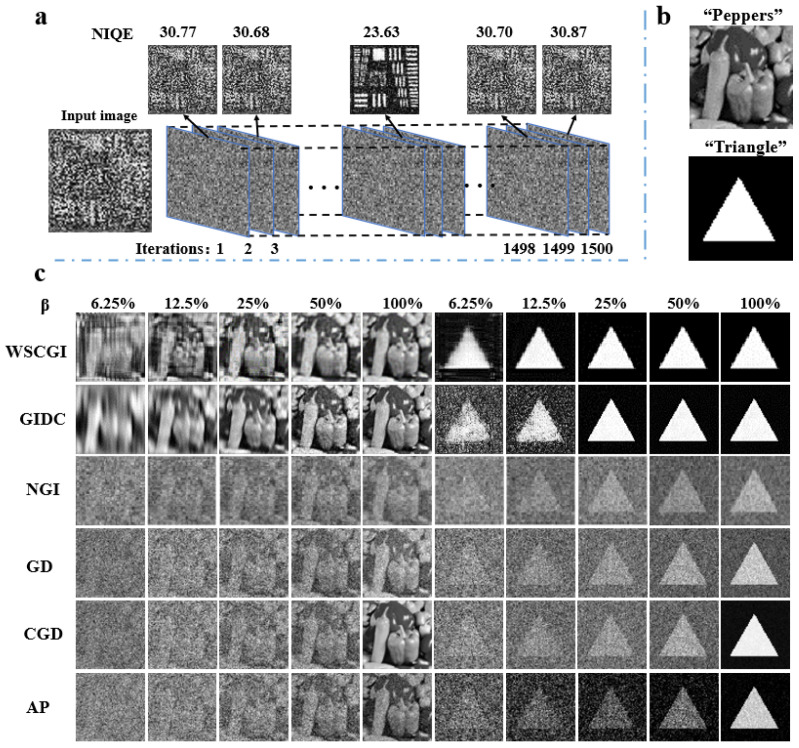
Results of numerically simulated reconstruction of an unobstructed object. (**a**) The selection method for WSCGI network results, which aims to determine the optimal solution among 1500 output results. The selection process is based on NIQE, with lower NIQE scores indicating higher image quality. (**b**) Original object; (**c**) output results when β = 6.25%, β = 12.5%, β = 25%, β = 50%, β = 100%, using various methods including GI, normalized ghost imaging (NGI), gradient descent (GD), conjugate gradient descent (CGD), alternating projection (APGI), GIDC [36].

The results are summarized in Figure 3. A lower NIQE value in Figure 3a indicates a higher image quality. In Figure 3c, we show the results obtained using normalized ghost imaging (NGI), where random speckle patterns are used as illumination modes, gradient descent (GD), and conjugate gradient descent (CGD), which are methods of compressive sensing ghost imaging, and alternating projection (APGI), which involves alternating projections in ghost imaging. It is evident from the results that even at a sampling rate of 100%, the results of NGI, DG, and APGI are not ideal. There is noise in the background, and the image is blurry, consistent with the previous discussion. It is worth noting that the image quality of CGD also significantly deteriorates with the decrease in the sampling rate. The imaging effect of WSCGI is similar to that of GIDC. However, our method has lower noise in the image at lower sampling rates. Although WSCGI is not the optimal solution among the methods, it can still obtain high-quality images at low sampling rates. Moreover, WSCGI is mainly used to obtain images of occluded objects, so this part of the experiment cannot fully demonstrate the full capabilities of this method. To quantitatively evaluate the results obtained through different methods, we calculated the structural similarity index (SSIM) for each reconstructed image relative to its corresponding reference standard. Table 1 shows the SSIM values.

Next, we simulated a ghost imaging experiment to restore occluded objects. After analyzing the data, we decided to set the sampling rate at 25% since it produced satisfactory results using less data and less computational time.

**Figure 4:**
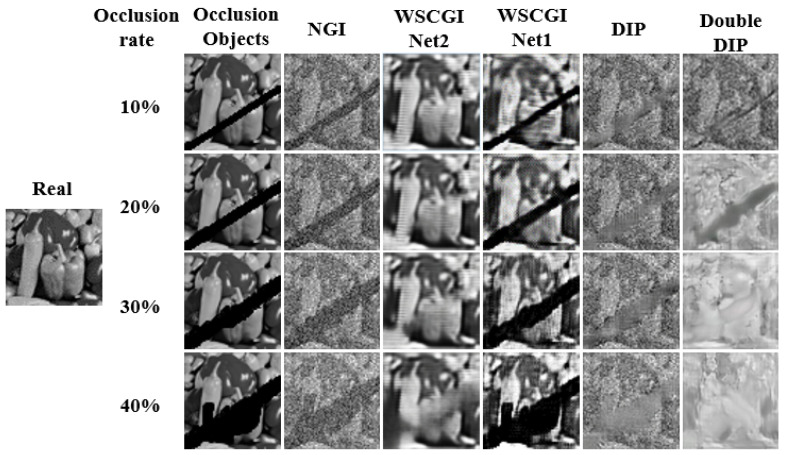
Comparison among the different reconstruction methods for various occlusion rates. DIP [45] and double DIP [46] are two self-supervised deep learning methods for image deocclusion. The occlusion rate is the ratio of the occluded area to the area of the object.

**Figure 5:**
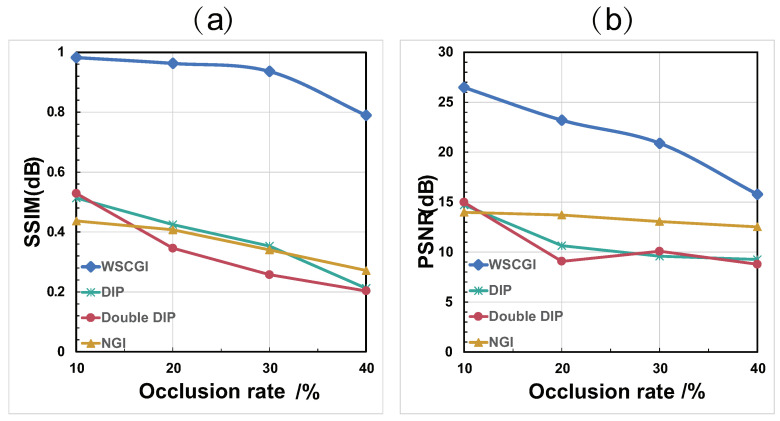
Objective evaluation of reconstruction results f0r different occlusion rates. (**a**) SSIM curve. (**b**) PSNR curve.

In Figure 4, the simulation results for the reconstruction of occluded objects for occlusion rates ranging from 10% to 40% are presented. It is apparent that WSCGI outperforms NGI, DIP, and double DIP in terms of image quality. WSCGI effectively restores the images of partially occluded objects at 20% or lower occlusion, partially restores them at 30% occlusion with some blurring, and exhibits less noticeable recovery at 40% occlusion. To assess the imaging performance of WSCGI under different occlusion conditions, we conducted SSIM and PSNR analyses. The SSIM and PSNR curves for different occlusion rates are shown in Figure 5a,b, respectively. Our quantitative results indicate that WSCGI performs better in recovering images with low occlusion rates, while its performance decreases with increasing occlusion rate. Clearly, recovering images with larger occluded areas poses a challenge, which we plan to investigate in future work.

## 4. Optical Experiment

**Figure 6:**
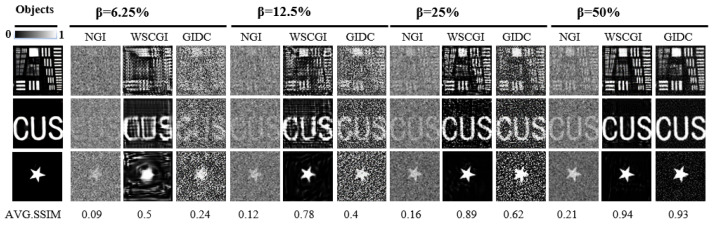
Experimental reconstruction by NGI and WSCGI. Each row represents the reconstruction results of the same object using different methods, while each column represents the results of different sampling rates using the same method.

We also conducted optical experiments, collecting data with the computational ghost imaging system illustrated in Figure 1. The size of the reconstructed images was set to 64 × 64 pixels. Here, we compared the performance of NGI, GIDC, and WSCGI at different sampling rates. The experimental results, shown in Figure 6, indicate that WSCGI successfully reconstructed the target object using only 256 measurements (β = 6.25%). Although the resulting image appears slightly blurry, it still retains the overall outline of the object. The image quality improved significantly as the sampling rate increased. In all test scenarios (different objects and β values), WSCGI outperformed NGI and GIDC in terms of visual appearance and quantitative evaluation metrics (especially SSIM). When β exceeded 25%, the SSIM was larger than 90%, which is consistent with our simulation results. Furthermore, the experimental results suggest thatnthe images reconstructed by NGI are compromised by noise-induced degradation, resulting in lower contrast.

**Figure 7:**
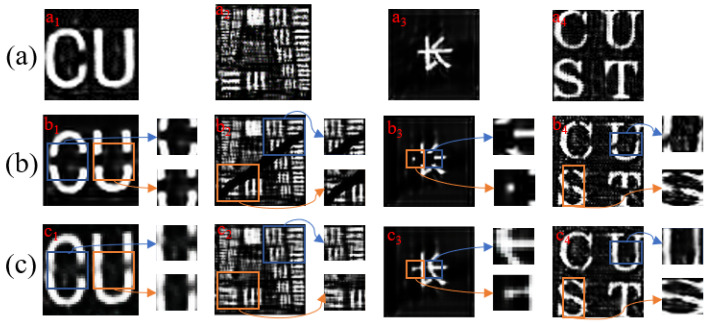
Experimental results obtained by partially occluding the target object. each arrow points to the corresponding area of local magnification in the box. (**a**) images processed by Net1 without local occlusion; (**b**) the partially occluded target, from left to right: horizontally occluded (Single layer letters), obliquely occluded (Resolution board), centrally occluded (Chinese characters), and multi-segment discontinuous occlusion(Double layered letters); (**c**) the image processed by the complete neural network.

Furthermore, we conducted experiments at a sampling rate of 25%, introducing black opaque stripes as occluders objects. After feeding the collected bucket detection signals and speckle signals into the neural network, the following experimental results were obtained:

Figure 7$b_{1}$ shows horizontally occluded letters, Figure 7$b_{2}$ represents the resolution map of diagonal occlusion, Figure 7$b_{3}$ shows Chinese characters occluded by a central square, and Figure 7$b_{4}$) represents multiple segments of discontinuous occlusion. We can observe that the restoration effect in Figure 7$c_{1}$ us not as satisfactory as that in Figure 7$c_{2}$,$c_{3}$, which may be due to the introduction of a relatively high occlusion rate in Figure 7. In Figure 7$b_{4}$, we also show multiple segments of discontinuous occlusion, using different sizes and angles of multiple segments of occlusion to occlude the target object. The results are shown in Figure 7$c_{4}$, which better verify the effectiveness of WSCGI in occlusion imaging. Overall, our experimental results are highly consistent with the simulation results. WSCGI is effective in imaging occluded objects, but there is still potential for improvement in high-occlusion scenes.

## 5. Conclusions

In this paper, we proposed an innovative approach termed WSCGI, which exploits deep learning to restore occluded objects by ghost imaging, without requiring large-scale datasets. WSCGI also exhibits the capability to achieve the high-quality restoration of partially occluded objects at low sampling rates. Using numerical simulations and optical experiments, the effectiveness and feasibility of WSCGI in practical applications were verified, offering new insights and solutions for the advancement of ghost imaging technology. Future work will focus on further improving the image restoration performance of WSCGI under high occlusion rates.

## Figures and Tables

**Table 1:** SSIM obtained with different GI reconstruction methods and at different sampling rates.

Object	“Peppers”						“Triangle”				
Method\β	6.25%	12.25%	25%	50%	100%		6.25%	12.25%	25%	50%	100%
WSCGI	0.66	0.79	0.89	0.91	0.96		0.97	0.98	0.98	0.98	0.98
NGI	0.09	0.10	0.11	0.12	0.12		0.09	0.14	0.18	0.29	0.36
GIDC	0.61	0.80	0.89	0.93	0.97		0.56	0.76	0.96	0.97	0.99
GD	0.26	0.30	0.46	0.63	0.81		0.12	0.17	0.22	0.37	0.58
CGD	0.22	0.30	0.46	0.63	0.81		0.12	0.17	0.22	0.37	0.98
AP	0.20	0.27	0.45	0.65	0.80		0.17	0.27	0.36	0.54	0.85

## Data Availability

The raw data supporting the conclusions of this article will be made available by the authors on request.
